# Knockdown of ApoL1 in Zebrafish Larvae Affects the Glomerular Filtration Barrier and the Expression of Nephrin

**DOI:** 10.1371/journal.pone.0153768

**Published:** 2016-05-03

**Authors:** Ahmed M. Kotb, Ole Simon, Antje Blumenthal, Silke Vogelgesang, Frank Dombrowski, Kerstin Amann, Uwe Zimmermann, Karlhans Endlich, Nicole Endlich

**Affiliations:** 1 Institute of Anatomy and Cell Biology, University Medicine Greifswald, Greifswald, Germany; 2 Institute of Pathology, University Medicine Greifswald, Greifswald, Germany; 3 Department of Nephropathology, Institute of Pathology, University Hospital Erlangen, Erlangen, Germany; 4 Department of Anatomy and Histology, Faculty of Veterinary Medicine, Assiut University, Assiut, Egypt; 5 Department of Urology, University Medicine Greifswald, Greifswald, Germany; Institut National de la Santé et de la Recherche Médicale, FRANCE

## Abstract

APOL1, a secreted high-density lipoprotein, is expressed in different human tissues. Genetic variants of *APOL1* are described to be associated with the development of end stage renal diseases in African Americans. In human kidney, APOL1 is mainly expressed in podocytes that are responsible for proper blood filtration. Since mice do not express ApoL1, the zebrafish is an ideal model to study the role of ApoL1. Injection of morpholinos against zApoL1 into zebrafish eggs and larvae, respectively, induces severe edema indicating a leakage of the filtration barrier. This was demonstrated in zApoL1 knockdown larvae by intravascular injection of fluorescently-labeled 10- and 500-kDa dextrans and by clearance of the vitamin D-binding protein from the circulation. Immunohistochemistry and RT-PCR revealed the reduction of nephrin, a podocyte-specific protein essential for blood filtration. Coinjection of human nephrin mRNA rescued the zApoL1 knockdown induced phenotype. Reduced APOL1 and nephrin levels were also found in biopsies of patients suffering from end stage renal diseases. Our results demonstrate that zApoL1 is essential for proper blood filtration in the zebrafish glomerulus and that zApoL1 affects the expression of nephrin.

## Introduction

Apolipoprotein L1 (*APOL1*) is one of six gene members of the apolipoprotein L family in humans that are clustered on chromosome 22. Two transcript variants of *APOL1* encoding for two isoforms have been identified. One isoform contains beside the typical functional domains of the APOL family—the MAD, BH3, PFD and SRA domain—a secretory signal domain (S), which confers resistance to Trypanosoma bruci [1–3]. Studies have shown that APOL1, a BH3-only lipid-binding protein, induces autophagy and apoptosis (Zhaorigetu et al. 2008). Recently, it has been published that three variants in the coding sequence of the *APOL1* gene are responsible for the development of different types of non-diabetic end stage renal disease (ESRD), like idiopathic focal segmental glomerulosclerosis (FSGS), HIVAN and hypertension-induced nephropathy in patients with African ancestry and Hispanics.

In many ESRD one specific epithelial cell in the glomerulus, the podocyte, is affected. Podocytes cover the outer aspect of the glomerular capillaries with interdigitating foot processes. Between these interdigitating foot processes, essential podocyte-specific proteins like the transmembrane protein nephrin form the slit diaphragm that is important for proper blood filtration. Slit diaphragm proteins are located in lipid rafts, cholesterol-enriched membrane domains that are important for the formation of multiprotein complexes. These multiprotein complexes can activate intracellular signaling cascades. Interestingly, it is suggested that APOL1, as an integral component of HDL particles, might be involved in cholesterol efflux from the cell modulating the lipid raft domains in the plasma membrane. Accordingly, APOL1 might play a role for the formation of specific signaling complexes in podocytes (Fornoni et al. 2014). Therefore, it is very likely that the expression of APOL1 plays a role for the cholesterol metabolism and in the regulation of autophagy. Both mechanisms have already been shown to contribute to podocyte dysfunction in glomerulopathies (Fornoni et al. 2014, Fougeray & Pallet 2015)

Since APOL1 is restricted to the genomes of humans and some primates like green African monkey and baboon [1,3–5] and is absent in the genome of dogs and rodents, the role of APOL1 has not been studied in a mammalian model until yet. Since the alignment of human APOL1 with the zebrafish genome showed, that the zebrafish expresses a protein with high homology to human APOL1 (Anderson et al. 2014), the zebrafish larva is a well-suited model to study the function of zApoL1 in a living organism. In this study we compared the renal expression of human APOL1 to zebrafish zApoL1 and studied the role of zApoL1 by knockdown (KD) of the protein with specific morpholinos.

## Material and Methods

### Zebrafish strains

Zebrafish wild type strain AB and the following transgenic stains were used (S1 Fig): ET (Tg (mitfa^w2/w2^; roy^a9/a9^; Tg(235.1wt1a:eGFP)) [6], CADE (Tg(**Ca**sper; l-fabp:**D**BP-**E**GFP)) [6], CET (mitfa^w2/w2^;roy^a9/a9^;Tg(-3.5fab10a:gc-EGFP)^lri500Tg^;Tg(wt1a:EGFP)^li4Tg^)). All zebrafish strains were raised, mated and maintained at 28.5°C, as previously described [7]. Embryos were kept and handled in E3 solution. We use zebrafish larvae 5dpf and below. The animal experiments are under guidelines of LAGUS Rostock and LALLF M-V/TSD, the use of biopsies were authorized by the ethic committee in Greifswald.

### Morpholino injection

Morpholinos (MOs) were designed and ordered by Gene Tools LCC (Philomath, OR, USA) as follows: zApol1 TBM (translation blocking morpholino): 5'-ATC GAA AGT CGT CCA GCC ATT CCA T-3'; zApol1 SBM exon2 (splice blocking morpholino) targeted to the exon2-intron2 junction: 5'-CCT TAT AAA AGG CCC ACC TGC ATG A-3'; zApol1 SBM exon6 (splice blocking morpholino) targeted to the exon6-intron6 junction: 5'-TAG GAT ATT ACA GTA CCT CTG CAA C-3'; *vivo*-zApol1 SBM exon2 sequence: 5'-CCT CTT ACC TCA GTT ACA ATT TAT A-3'; nephrin morpholino (translation blocking morpholino): 5'-CGCTGTCCATTACCTTTCAGGCTCC-3'. As a standard control we used CtrlMO and *vivo*-CtrlMO offered by Gene Tools. Morpholinos were diluted to a concentration of 10 mM. A volume of approximately 3 nl morpholino per zebrafish embryo was injected into two to four-cell stage fertilized eggs using a microinjector (Transjector 5246, Eppendorf). For *in vivo* KD, a volume of approximately 6 nl injection buffer was injected into the cardinal vein of 3dpf larvae.

### Synthesis of human Nephrin mRNA

The human plasmid encoding for Nephrin was a kind gift from Dr. Puneet Garg (University of Michigan, USA). The plasmid was digested with restriction enzymes and used as a template for the synthesis of mRNA (mMESSAGE mMACHINE kit; LifeTechnologies, AM1348). Morpholinos and mRNA (60 pg) were co-injected into one cell stage embryos. The following primers were used to detect the expression of human Nephrin mRNA in zebrafish larvae (2 dpf): Forward primer: ATTAACCCTCACTAAAGGGAAC; Reverse primer: GATAATAGGCAGTTGCTGGT.

### RT-PCR

RNA isolation of different organs (adult zebrafish and zebrafish larvae) was done with the RNeasy Plus Universal Mini Kit (Qiagen). For RNA translation the Superscript II Reverse Transcriptase (Invitrogen) was used. Gene transcription was detected using the following primers (forward—fw; reverse—rv): see S1 Table.

### Filtration assay

The procedure has been published recently by us [8]. Briefly, larvae were anesthetized with 0.1–0.5% tricaine (Sigma Aldrich) and injected with as a 1:1 mixture of 25 mg/ml Alexa fluor 647-conjugated 10 kDa dextran and 10 mg/ml FITC-conjugated 500 kDa dextran (Invitrogen) into the caudal vein of 3 dpf larvae using femtotips micropipettes (Eppendorf). The larvae were transferred to E3 medium for recovery. Three hours after injection the larvae were anesthetized again. After positioning of the larvae in 8% methylcellulose (in E3 medium), the vasculature and the pronephric tubules were imaged by a Leica TCS SP5 microscope (Leica Microsystems, Wetzlar, Germany) using a water immersion objective (HCX PL APO 20x/0.7). For the quantitative analysis of dextran clearance, image stacks of the dorsal aorta and the cardinal vein caudal of the swim bladder were recorded for both fluorescence channels at fixed time points after injection (3, 6, 10 and 24 hours). Between recordings, the larvae were transferred to E3 medium at 28°C for recovery.

### Immunohistology

For paraffin tissue sections, zebrafish larvae and organs were fixed in 2% PFA for 1 day, dehydrated in ascending concentrations of ethanol in PBS (25%, 50%, 70%, and 100%) and transferred in xylene (100%) for 5 min before embedding in paraffin (60°C) overnight. With a rotational microtome (Leica SM 2000 R) sections of 5 μm were cut, incubated in ethanol (100%, 70%, 50%, and 25%) and stained with HE (Merck, Germany). For cryosections larvae were fixed in 2% PFA for 2 h followed by an overnight incubation in 30% saccharose at 4°C. After embedding the larvae in Tissue-Tek (Sakura, Staufen, Germany), sections (20 μm, 60 μm) were cut using a Leica CM 1950 microtome and stained with antibodies as described [7,9].

The following antibodies were used: anti-human synaptopodin (Progen, Germany), anti-human APOL1 (Proteintech, USA), anti-zebrafish ApoL1 (zApoL1 (276–289): CKDSHELKNGAKSEF, Innovagen, Sweden) and anti-zebrafish nephrin (zNephrin, Innovagen, Sweden). As secondary antibodies, Cy3-conjugated anti-rabbit and Cy3-conjugated anti-mouse antibodies (Dianova, Hamburg, Germany) were applied. Alexa Fluor 546-conjugated phalloidin (Invitrogen) and Hoechst 33342 (Sigma-Aldrich) were used to visualize F-actin and nuclei, respectively.

### Transmission electron microscopy

Zebrafish larvae were fixed with 1.5% glutaraldehyde and 0.5% paraformaldehyde as described [7] and embedded in LR White (Plano, Wetzlar, Germany). Ultrathin sections were cut with the Ultracut UCT microtome (Leica GmbH, Germany) and stained with the primary antibody against zApoL1 first followed by an incubation with the secondary antibody conjugated with colloidal gold (10 nm). Sections were contrasted with 5% uranyl acetate and lead citrate [10] and examined with a JEOL JEM 1011 electron microscope (JEOL, Tokyo, Japan).

### Imaging

Zebrafish larvae were controlled with a Stemi SV11 stereomicroscope (Carl Zeiss Microimaging, Jena, Germany) with the use of a 2.5x air objective (2.5x/0.075 Plan Neofluar). HE stained sections were imaged with a Olympus BX50 microscope. Confocal microscopy was performed with a Leica TCS SP5 microscope (APO 20x, APO 60x). For *in vivo* observation, zebrafish larvae were embedded in low-melting 0.8% agarose (Biozyme, Germany) and positioned in a dorsal-up position by an eyelash pencil as described [9]. Images and movies were taken with a Zeiss LSM7MP multiphoton microscope (Carl Zeiss Microimaging, Jena, Germany) using 20x water immersion objectives.

### Human biopsies

Human kidney samples were frozen in liquid nitrogen immediately after resection. The samples were fixed in 2% PFA overnight, dehydrated in ascending concentrations of ethanol in PBS (25%, 50%, 70%, and 100%) and transferred in xylene (100%) for 5 min before embedding in paraffin (60°C) overnight. With a rotational microtome (Leica SM 2000 R) sections of 5 μm were cut, incubated in ethanol (100%, 70%, 50%, and 25%) and stained with anti-human synaptopodin (Progen, Germany), anti-human APOL1 (Proteintech, USA) and anti-human nephrin (Progen, Germany) antibody. The anti-zebrafish ApoL1 antibody was designed, produced and purified by Innovagen (Lund, Sweden).

## Results

### zApoL1 is widely expressed in zebrafish organs

**Fig 1:**
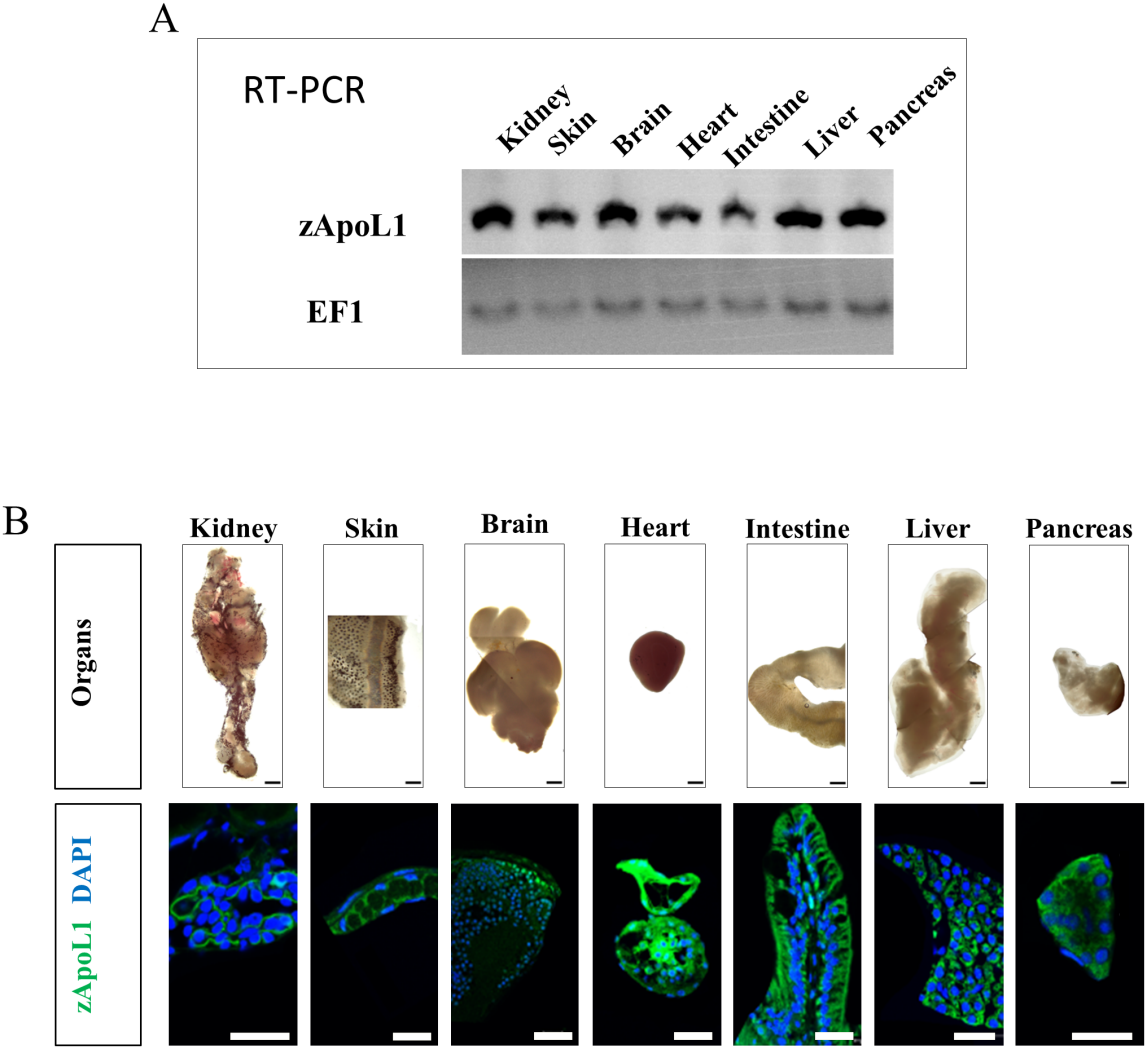
zApoL1 expression in different zebrafish organs. The expression of zApoL1 was determined in different organs like the kidney, skin, brain, heart, intestine, liver and pancreas if adult zebrafishs by RT-PCR in panel A and by immunohistochemistry in panel B. As a housekeeping gene, the elongation factor 1 (EF1) was used. Scale bars in panel B represent 500 μm (top) and 20 μm (bottom).

To study the localization and function of ApoL1 *in vivo* we used the zebrafish model. Expression of zApoL1 (ENSDARG00000007425) was verified using total RNA from different zebrafish organs like kidney, skin, brain, heart, intestine, liver and pancreas. As shown in Fig 1A, all organs of the adult zebrafish showed a strong signal for zApoL1 mRNA. To identify the localization of zApoL1 we stained sections of the organs with a specific antibody against zApoL1 (Fig 1B). In the zebrafish mesonephros a strong signal was found exclusively in the glomerulus.

### zApoL1 is expressed in podocytes and endothelium in the zebrafish pronephros

**Fig 2:**
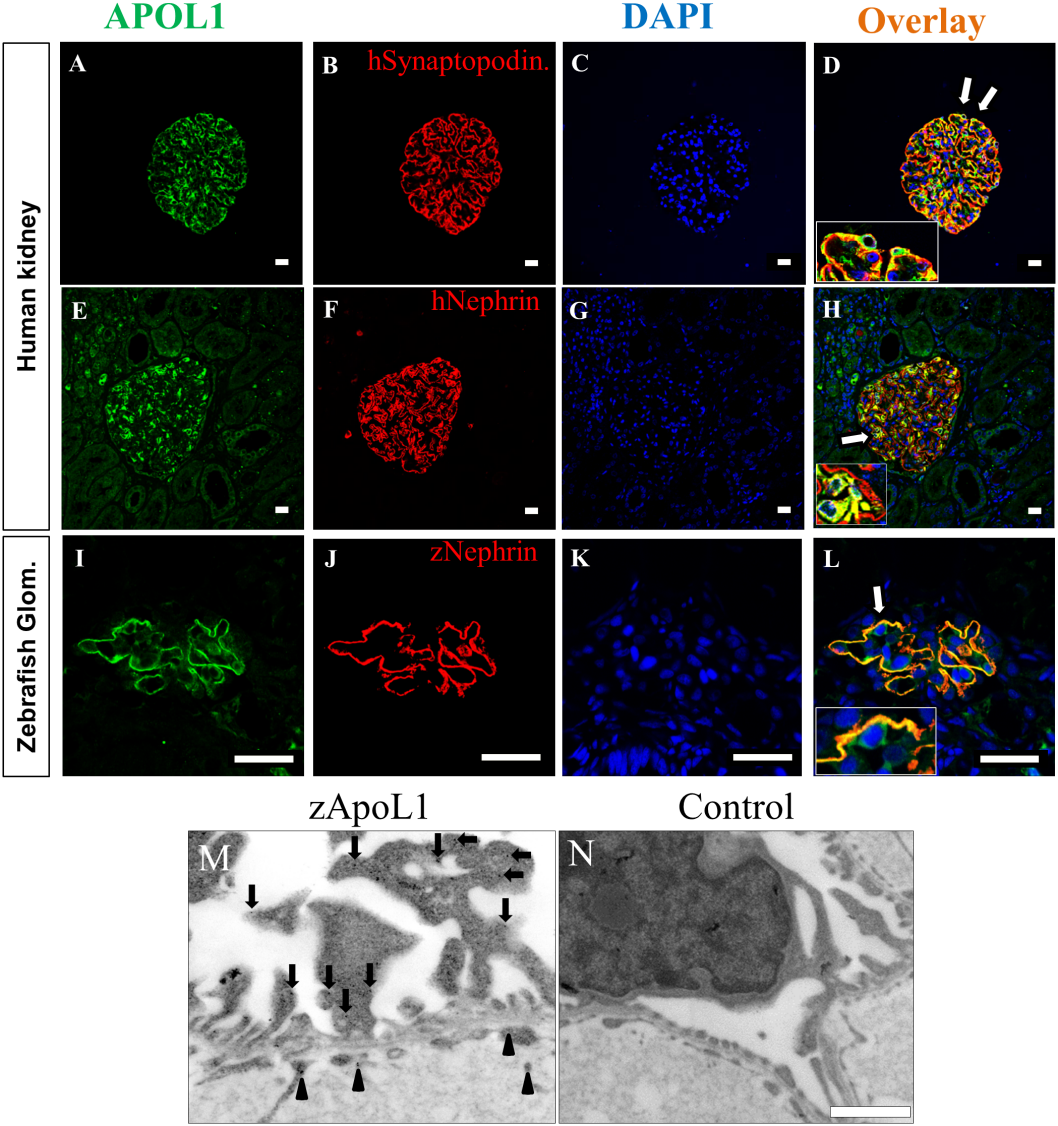
APOL1 expression in human and zebrafish glomeruli. The expression of APOL1 in podocytes is shown by double staining with an antibody against APOL1 and an antibody against the podocyte-specific proteins synaptopodin and nephrin (A-H). The regions in panel D and H labeled by arrows are shown in higher magnifications (insets). Similar to the human kidney, zApoL1 colocalizes with zNephrin in the zebrafish glomerulus (L). Panel M shows the localization of zApoL1 in podocytes (red circles) and in endothelial cells (green circles) by immunoelectron microscopy. Panel N shows the control reaction (secondary antibody only). Scale bars represent 20 μm (A-L) and 1 μm (M-N).

To further investigate the localization of zApoL1 in the kidney we stained tissue of humans and zebrafish larvae with antibodies against APOL1 (hAPOL1, zApoL1). As shown in Fig 2A, 2E and 2I an intense signal was detected in the glomerulus of humans as well as zebrafish larvae (4 dpf). Colocalization studies with antibodies against the podocyte-specific proteins nephrin and synaptopodin showed that APOL1 is strongly expressed in human podocytes (Fig 2A–2H). In contrast to the localization of synaptopodin that is restricted to the foot processes of podocytes, APOL1 was also found in the cell body of human podocytes (insets in Fig 2D). Furthermore, we co-stained sections of zebrafish larvae with zApoL1 and zNephrin antibodies. Fig 2I–2L shows that both proteins are colocalized specifically in podocytes. Higher magnifications of glomerular capillaries revealed that zApoL1 is also expressed in glomerular endothelial cells (inset in Fig 2L) however to a lesser extent compared to podocytes.

Additionally, we studied the localization of zApoL1 in podocytes by immunoelectron microscopy. We observed that zApoL1 is localized in major processes and foot processes of podocytes as well as in endothelial cells (Fig 2M). For better visualization we have labeled the gold particles with green and red circles. As a negative control, we used sections that were stained with the secondary antibody only (Fig 2N).

### zApoL1 KD induces pericardial edema formation and an increase of Bowman’s space in the glomerulus

**Fig 3:**
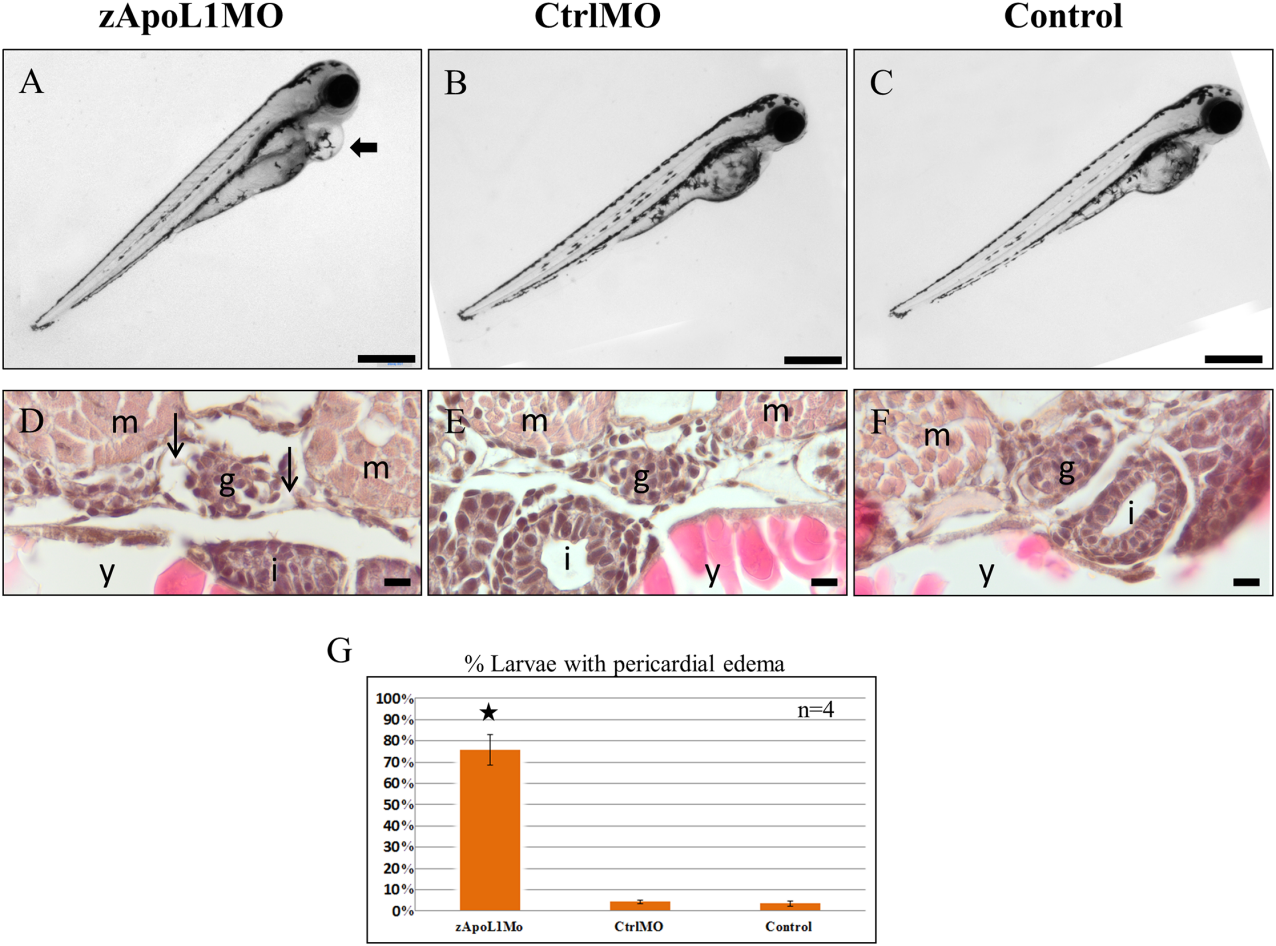
zApoL1 KD induces pericardial edema formation. Zebrafish larvae (4 dpf) developed edema (arrow in A) after the KD of zApoL1 in contrast to CtrlMO and untreated larvae (B, C). Paraffin tissue sections of the larvae showed enlarged Bowman’s space (arrows in D) due to the knockdown of zApoL1 compared to CtrlMO and untreated larvae (E, F). g—glomerulus, m—myotome, i—intestine, y—yolk sac. Panel G shows that more than 70% of the larvae developed edema in response to KD of zApoL1. Data are means±SD of 4 experiments on a total of 100 larvae. Scale bars represent 500 μm (A-C) and 10 μm (D-F). We have used t-test for statistical analysis.

To study the influence and the function of zApoL1 *in vivo* we knocked down the expression of zApoL1 by the use of morpholinos. Therefore, we generated 3 different morpholinos that are described in the S2A–S2C Fig. Three to four days after injection of the morpholinos, zebrafish larvae developed severe pericardial edema (arrow in Fig 3A). In contrast, the control morpholino injected larvae (CrtlMO; Fig 3B) and the untreated larvae (Control; Fig 3C) developed normally. Since 3 different morpholinos gave the same results we used often only zApoL1 TBM in the following experiments. KD of zApoL1 was verified by RT-PCR (S2D Fig).

We found that 76% of zApoL1MO larvae (100 larvae, n = 4) developed pericardial edema at 4 dpf (Fig 3G) without any cardiac defects. Since edema formation is a hallmark of a leaky glomerular filtration barrier, we studied the effect of the zApoL1 KD on the glomerulus by using H&E stained sections of zebrafish larvae (4 dpf). While the glomeruli of CrtlMO and Control larvae were developed normally (Fig 3E and 3F), Bowman’s space was increased (arrows in Fig 3D) after KD of zApoL1.

### zApoL1 KD changes the morphology of the glomerulus

**Fig 4:**
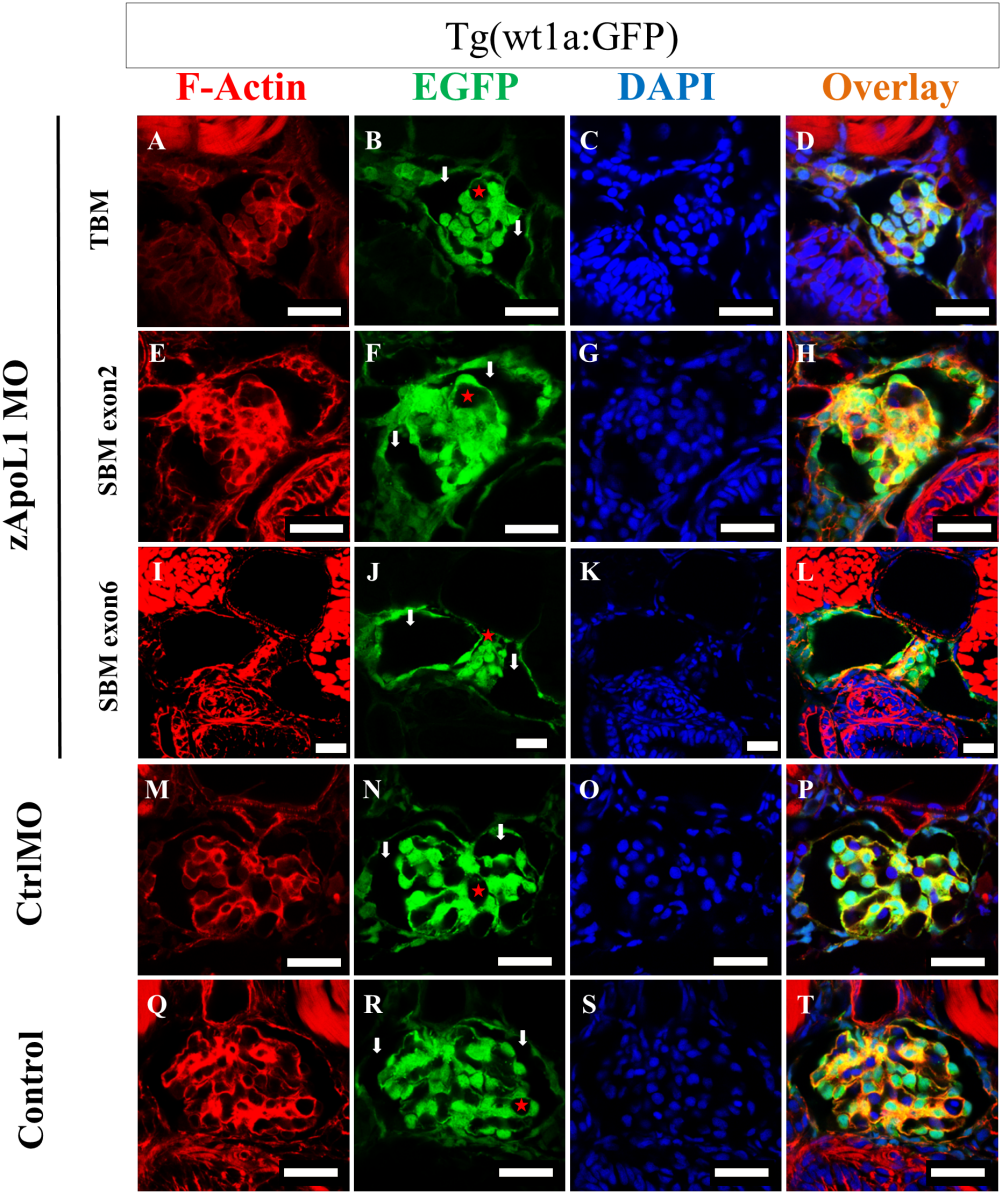
zApoL1 KD changes the morphology of the glomerulus. zApoL1 was knocked down in larvae expressing EGFP in podocytes by zApoL1 TBM (A-D), zApoL1 SBM exon2 (E-H) and zApoL1 SBM exon6 (I-L) morpholinos. Paraffin sections of the larvae were stained with Alexa 546-phalloidin for F-actin and DAPI for the nuclei. The KD with three different morpholinos against zApoL1 resulted in a similar phenotype, comprising dilation of Bowman’s space (arrows in B, F, J), collapse and malformation of the glomerular tuft (asterisks in B, F, J). CtrlMO and Control larvae are shown in panel M-T. Scale bars represent 20 μm.

To study the morphology of the glomerulus we analyzed histological sections of zebrafish larvae expressing EGFP in podocytes. Further, to visualize the F-actin cytoskeleton we stained with Alexa-546 phalloidin. As shown in (Fig 4M–4P and 4Q–4T), CrtlMO as well as Control larvae showed a normally developed glomerular tuft covered by green-fluorescent podocytes. In contrast, KD of zApoL1 resulted in significant changes of the morphology of the glomerulus. Independent of the morpholinos that were used (zApoL1 TBM, SBM exon2, *vivo*-SBM exon2 and SBM exon6), Bowman’s space became dilated (arrows in Fig 4B, 4F and 4J) after zApoL1 KD. Moreover, the formation of the glomerular tuft appeared to be significantly disturbed, and the glomerular capillaries were collapsed. Movie showed 3-D reconstructions of glomeruli of zApoL1MO (S1 Movie) and CrtlMO larvae (S2 Movie).

### The phenotype of zApoL1 KD is not due to developmental effects

To find out whether glomerular morphology was changed by KD due to developmental effects we injected *vivo*-morpholinos into the cardinal vein of zebrafish larvae (3 dpf). Three hours after injection of the *vivo*-zApoL1MO, pericadial edema was observed (S3A Fig). In contrast, the injection of control *vivo*-morpholinos (*vivo*-CrtlMO) showed no edema formation (S3B Fig). Histological sections of *vivo*-zApoL1MO treated larvae showed the same phenotype as the zApoL1MOs treated ones (S3C–S3H Fig).

### In vivo observation of podocytes in *v*-zApoL1MO larvae

With the aid of a two-photon microscope (2PM) it is possible to observe the glomerulus and podocytes in zebrafish larvae *in vivo* [9]. After injection of *vivo*-zApoL1MO we studied the dynamics of living podocytes. For this experiment we used the zebrafish strain ET that expresses EGFP in podocytes and lacks pigmented cells. Five hours after injection of *vivo*-zApoL1MO into the cardinal vein, z-stacks were recorded every 10 min, and glomeruli were reconstructed in 3-D (S3 Movie). Time lapse experiments showed that the glomerular morphology changed during the observation time of 6 hours in *vivo*-zApoL1MO larvae in contrast to *vivo*-CtrlMO larvae (S4A–S4H Fig and S4 movie). By 2PM we thus observed an increase of Bowman’s space in agreement with the results obtained by zApoL1MO injection into fertilized eggs.

### zApoL1 affects the glomerular filtration barrier of zebrafish larvae

**Fig 5:**
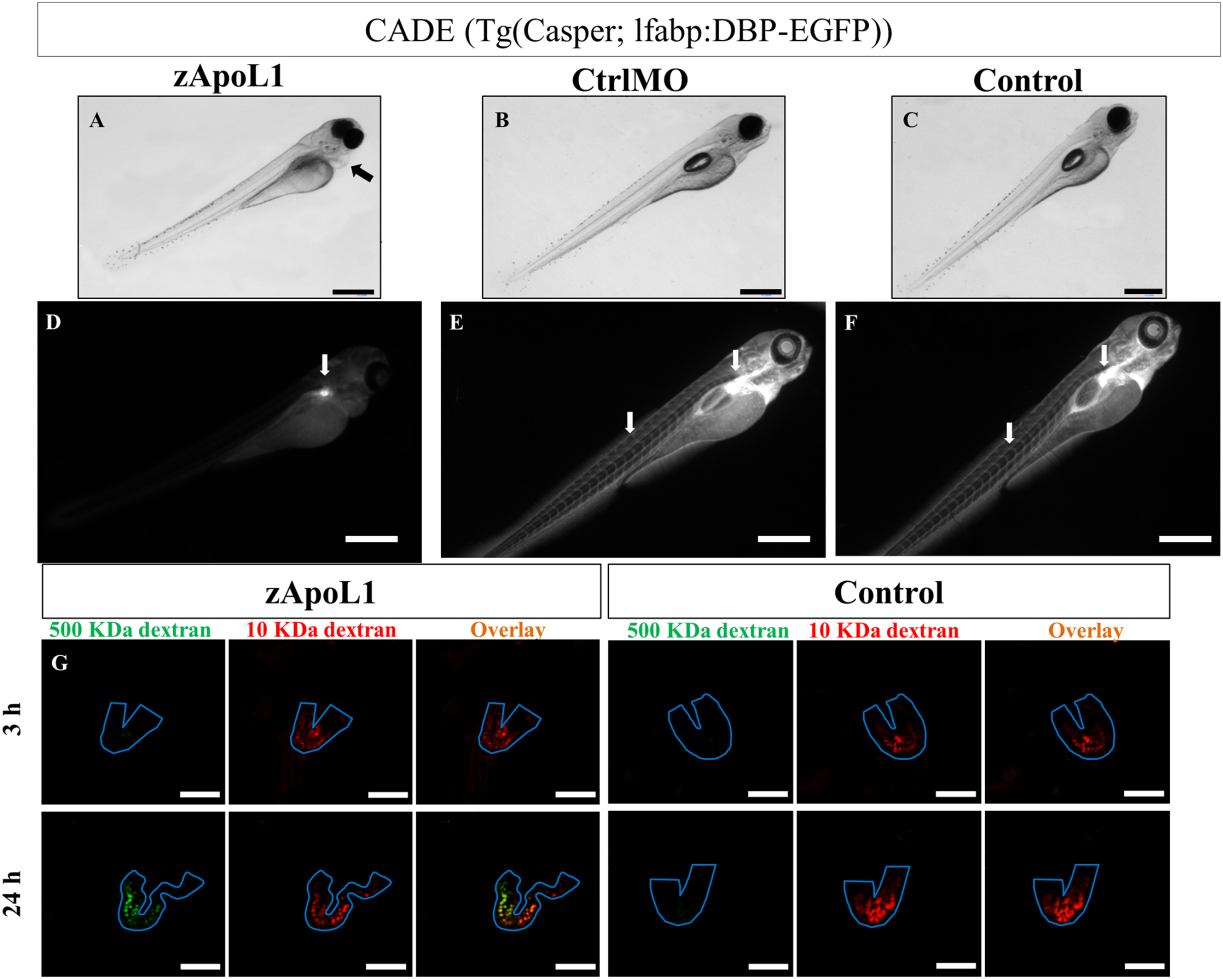
zApoL1 affects the glomerular filtration barrier of zebrafish larvae. After KD of zApoL1, larvae (CET strain) developed edema (arrow in A), a hallmark of a leaky glomerular filtration barrier. The loss of EGFP-vitamin D-binding protein from the blood resulted in a marked decrease of the fluorescence in the blood vessels (D). Only the fluorescence of the podocytes remained (arrow in D). In contrast the control larvae with an intact filtration barrier showed an intense staining of the vessels and the pronephric glomerulus (arrows in E, F). Alexa647-conjugated 10 kDa dextran and FITC-conjugated 500 kDa dextran were injected into the cardinal vene of the larvae (3 dpf). 3 and 24 h after injection, the fluorescence in the pronephric tubules (outlined in blue in G) was determined. Only zApoL1MO larvae showed a green fluorescence in the tubules at 24 h, indicating leakage of 500 kDa dextran through the glomerular filtration barrier. The control larvae showed only the red fluorescence of the filtered 10 kDa dextran. Scale bars represent 500 μm (A-F) and 50 μm (G).

For rapid screening of the glomerular filtration barrier, we generated a zebrafish strain named CET by breeding (cf. S1 Fig). Under healthy conditions the blood as well as the glomerulus of CET larvae fluoresce in green (arrows in Fig 5E and 5F). After the glomerular filter became leaky due to KD of zApoL1, the EGFP-labeled protein passed the filtration barrier and the intravascular fluorescence disappeared (Fig 5D). Wt1a-promoter driven EGFP fluorescence in podocytes remained unchanged (arrow in Fig 5D).

To quantify this defect we have already studied the filtration of a 1:1 mixture of Alexa Fluor 647-conjugated 10 kDa dextran and FITC-conjugated 500 kDa dextran that was injected into the cardinal vein caudal of larvae (4 dpf) in dependence of zApoL1 [8]. We found that the fluorescence of the FITC-conjugated 500 kDa dextran was completely lost in the blood vessels of zApoL1MO larvae after 24 hours (Fig 5G) in contrast to the control larvae. To confirm that the 500 kDa dextran was filtered and did not disappear into the interstitium we imaged the tubules of the pronephros. Endocytosed 500 kDa FITC-dextran appeared in the tubules (labeled in blue in Fig 5G) in a time-dependent way in zApoL1MO larvae only. This underlines the leakiness of the glomerular filtration barrier after KD of zApoL1.

### zApoL1 KD changes nephrin expression and podocyte foot process morphology

**Fig 6:**
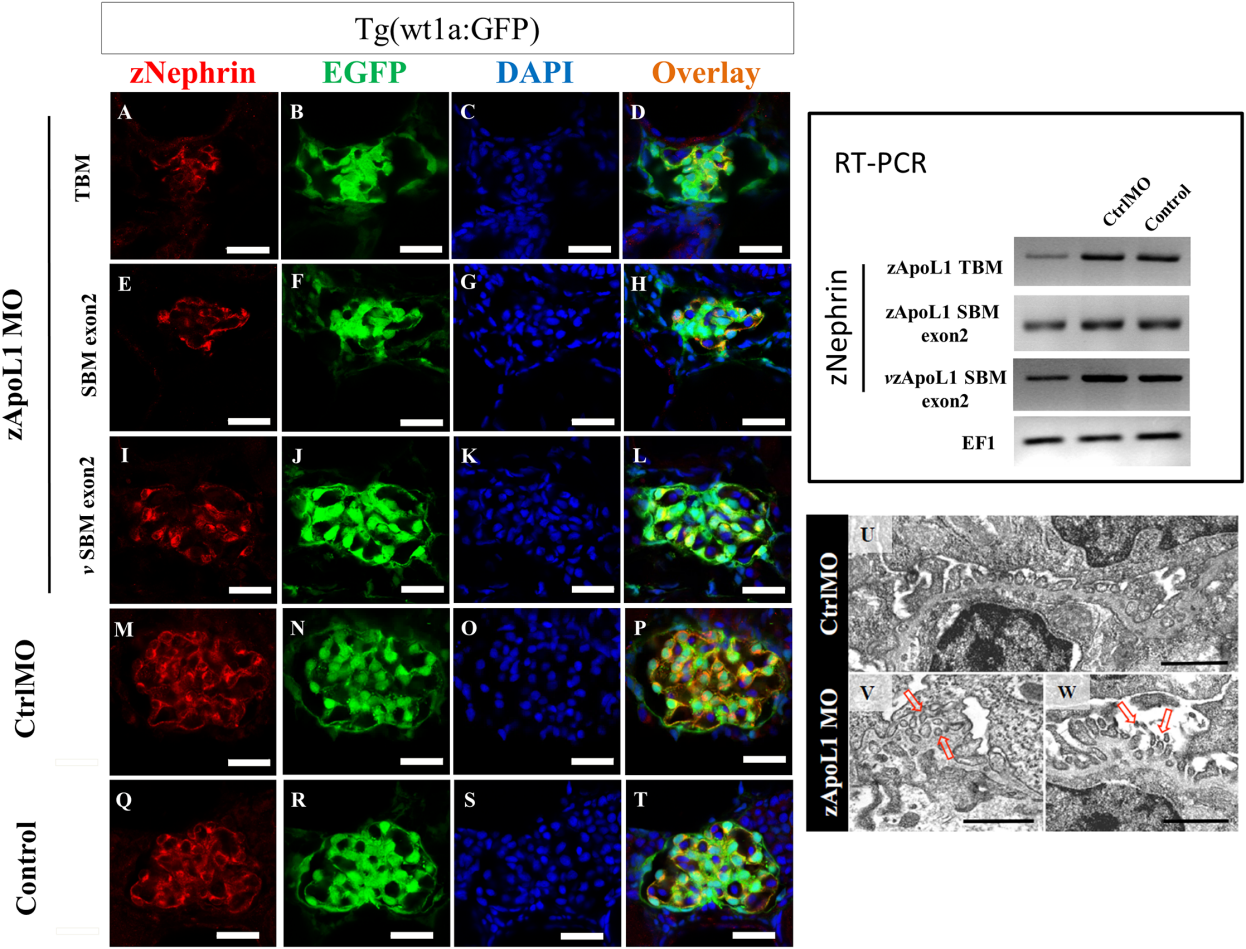
zApoL1 KD reduces the expression of nephrin. The expression of nephrin was significantly reduced in podocytes (green) after zApoL1 KD: zApoL1 TBM (A-D), zApoL1 SBM exon2 (E-H) and vivo-zApoL1 SBM exon2 (I-L) in contrast to CtrlMO (M-P) and untreated larvae (Q-T). RT-PCR shows a marked decrease of nephrin mRNA expression after zApoL1 knockdown compared to CtrlMO and control larvae. The elongation factor 1 (EF1) was used as a housekeeping gene. The right panel shows one representative experiment out of three. Scale bars represent 20 μm. (U) CtrlMO larvae have nicely developed foot processes in contrast to zApoL1 larvae that showed effacement of foot processes at some areas (V). Furthermore, the foot processes of zApoL1 KD larvae are sometimes surrounded by GBM (arrow in V) and developed microvilli-like structures protruding in the urinary space, respectively. Scale bar represents 1 μm.

Immunostaining with a specific antibody for zebrafish nephrin revealed that the expression of nephrin was significantly decreased in podocytes of zApoL1 KD larvae (Fig 6A–6L) in contrast to podocytes of the control larvae (Fig 6M–6T). This result was found with all 3 morpholinos against zApoL1 and was confirmed by RT-PCR (right panel of Fig 6). Electron microscopy displayed partial effacement of podocyte foot processes and the formation of microvilli protruding into the urinary space (right panel of Fig 6).

### Expression of APOL1 and nephrin is reduced in biopsies of patients with CKD

**Fig 7:**
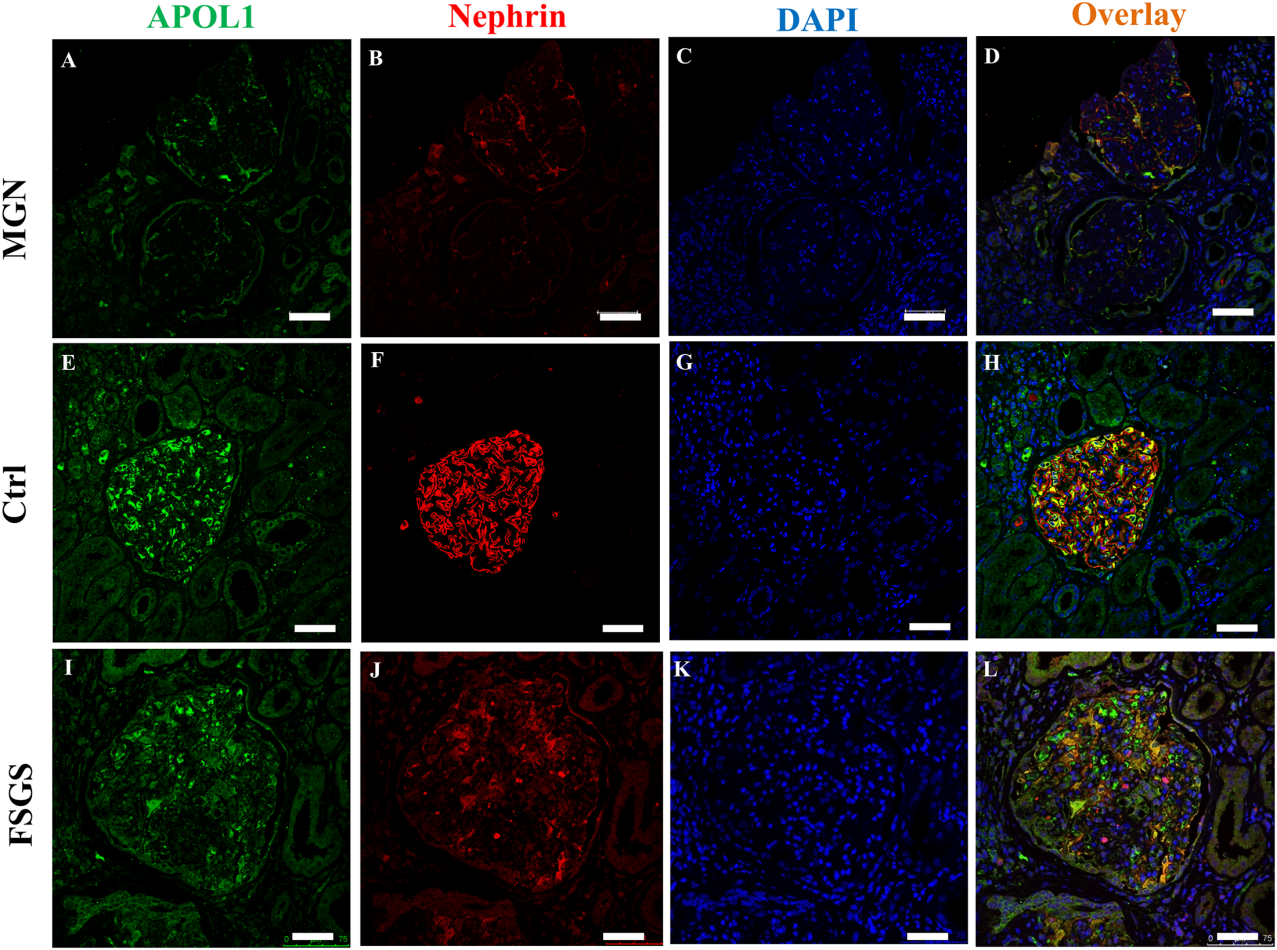
APOL1 and nephrin expression in kidney biopsies. Tissue sections of biopsies of patients suffering from membranous glomerulonephritis (MGN) and focal segmental glomerulosclerosis (FSGS) stained with antibodies against APOL1 and nephrin showed a marked decrease of APOL1 (A, I) as well as nephrin (B, J) in podocytes. Scale bars represent 20 μm.

To identify whether APOL1 protein expression is affected in human kidney disease, we stained human kidney sections against APOL1 and the specific podocyte marker nephrin. Kidney sections of membranous glomerulonephritis (MGN) and focal segmental glomerulosclerosis (FSGS) showed a marked decrease of the APOL1 expression (Fig 7A and 7I). Expression of nephrin was concomitantly decreased in podocytes with reduced levels of APOL1 (Fig 7B and 7J).

### zApoL1 KD can be rescued by nephrin mRNA injection into zebrafish larvae

**Fig 8:**
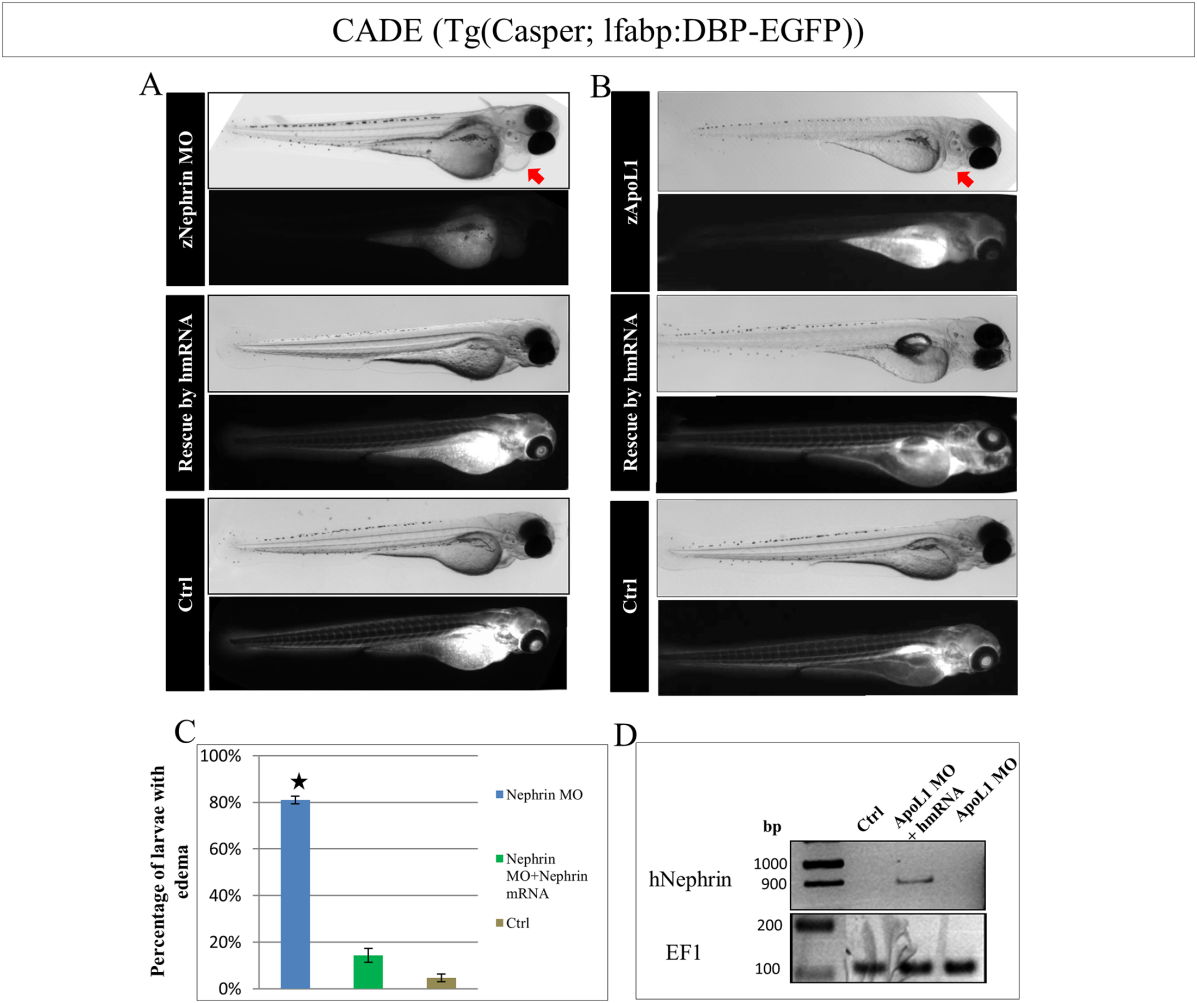
zApoL1 KD rescue in zebrafish larvae by human Nephrin mRNA. The rescue of the nephrin KD by human Nephrin mRNA (hmRNA) resulted in zebrafish larvae (*CADE*) without edema and with an intact filtration barrier (A). The KD of zApoL1 was rescued by co-injection of hmRNA resulting in zebrafish larvae without any phenotype (B). C shows the percentage of larvae with an edema after nephrin KD and rescue by hmRNA. The expression of the human mRNA was verified by RT-PCR as shown in D. We have used t-test for statistical analysis.

Since a reduction of ApoL1 in zebrafish and humans is associated with a reduction of nephrin we were interested to see whether the zApoL1 MO induced phenotype can be rescued by the presence of nephrin mRNA. As shown in Fig 8A, the nephrin KD (ENSDARG00000060758) in zebrafish larvae can be rescued by the injection of human nephrin mRNA (hmRNA). The knockdown of zApoL1 KD which induced severe pericardial edema formation as well as a leakage of the filtration barrier identified by the reduction of the blood fluorescence in the zebrafish larvae *Cade*, was rescued significantly by the co-injection of hmRNA (Fig 8B and 8C). The expression of the hmRNA in zebrafish larvae was verified by RT-PCR (Fig 8D). These results indicate a functional interaction between the ApoL1 and nephrin expression *in vivo*.

## Discussion

Two independent groups discovered an association between chronic kidney disease and the two coding variants G1 and G2 in the *APOL1* gene [2,11]. These two variants are located at the membrane-addressing domain (MAD) and the SRA-binding domain (SRA) at the C-terminus of APOL1 [12–14]. Several studies have confirmed that the *APOL1* variants are important risk factors for the development of focal segmental glomerulosclerosis and other forms of chronic kidney disease in African Americans and Hispanics [11,15]. However, direct experimental evidence of the physiologic role of APOL1 in podocytes and of the pathogenic mechanisms of the APOL1 variants in the development of chronic kidney disease is still lacking and animal models have not been described so far.

To study the function of APOL1 in vitro, Lan et al. used cultured human podocytes [16]. The expression of the APOL1 variants in cultured podocytes induced swelling and reduced the viability as compared with wild-type APOL1. These effects seemed to be caused by an increased permeability of lysosomal membranes and an APOL1-induced disruption of the F-actin cytoskeleton [16]. However it is not clear whether the effects are caused by a gain or a loss of function.

Since an ortholog of *APOL1* does not exist in mice, the mouse offers only limited possibilities to study the role of APOL1 in vivo. To circumvent this problem, Thomson and colleges expressed G1 and G2 by hemodynamic gene delivery (HGD) in mice [17]. They found that these HGD mice develop hepatic necrosis, especially if transfected with the G1 variant. However, kidney injury was not observed in this model. Obviously further model organisms are needed to study the in vivo function of APOL1 and its variants.

In the present study, we used the zebrafish as a model organism. Although zebrafish larvae have a simplified kidney, called pronephros, the glomerulus contains the same cell types (endothelium, mesangial cell and podocyte) as humans and mice [18,19]. Furthermore, podocyte function in the zebrafish pronephros relies on the same essential proteins (e.g. nephrin or podocin), as it is the case in humans and mice [20–22]. Furthermore, the zebrafish larvae used in our experiments were translucent and therefore it was possible to study the pronephric glomerulus in vivo by two-photon microscopy [9].

Zebrafish ApoL1 (zApoL1) bears significant homology to human APOL1, and it contains the MAD and SRA domains. Since zebrafish possess only one *ApoL* gene, the role of ApoL1 can be studied in the absence of functional redundancy, which might be mediated by further *ApoL* genes. Therefore, the zebrafish model provides relevant insight regarding the function of APOL1 in a living organism.

In accordance with recent studies [4,23], we found that zApoL1 is expressed in podocytes and to some extent in endothelial cells (Palmer et al. 2013) in human biopsies as well as in the glomerulus of zebrafish larvae. After injection of morpholinos against zApoL1 to knockdown the expression of zApoL1, the larvae developed severe pericardial edema, a hallmark of a perturbed kidney function. Utilizing our novel transgenic line CET, which expresses GFP-labeled Vitamin D-binding protein in the blood, we were able to show that the filtration barrier becomes leaky after KD of zApoL1. This finding was corroborated by accumulation of intravenously injected dextran in the pronephric tubule of zApoL1 treated larvae. Recently, we have obtained similar results by measuring the clearance of intravenously injected, fluorescently labeled 70 and 500 kDa dextrans from the blood of living zebrafish [8]. KD of zApoL1 was further associated with changes in glomerular morphology. Using *vivo*-morpholinos and two-photon microscopy, we could follow these morphological changes of the glomerulus in response to zApoL1 knockdown *in vivo* over time. Thus, our present study provides the first evidence that the loss of APOL1 can cause a glomerular phenotype. Since podocytes are mainly affected in ESRD it is very likely that zApoL1 of the endothelium plays a minor part during the development of glomerulopathies.

By immunohistological studies it became obvious that the changes in glomerular morphology are accompanied by a decrease in nephrin expression in podocytes. Furthermore, a co-regulation of APOL1 and nephrin was observed in biopsies from patients suffering from MGN or FSGS. By co-injection of human nephrin mRNA with zApoL1 MO we were able to show that the zApoL1 KD induced phenotype can be rescued by human nephrin mRNA suggesting a functional interaction between ApoL1 and nephrin. Interestingly, Anderson and coworkers have recently shown that there is a possible link between the expression of ApoL1 and Myh9, a protein responsible for an increased risk of CKD [24].

Surprisingly, an *APOL1* null individual from a small rural village in central India does not have glomerulosclerosis [25]. However, this observation is not in contradiction to an essential function of zApoL1 in zebrafish, since penetrance in humans may be incomplete necessitating the examination of more *APOL1* null individuals. Furthermore, humans possess additional *APOL* genes (*APOL2*, *3*, *4*, *5* and *6*) that might compensate for the loss of *APOL1*. In support of overlapping functions of APOL proteins, Smith et al. nicely showed that not all primate species express the complete collection of *APOL* genes [5]. For example, chimpanzees express only *APOL2*, *3*, *5* and *6*. One candidate that may share functions with APOL1 is APOL6, which is expressed in primates and in mice as well. Zhaorigetu et al. demonstrated that, similarly to APOL1, APOL6 is involved in autophagy and apoptosis [26].

In summary, the present study demonstrates that zApoL1 plays an important role in the glomerular biology of the zebrafish pronephros. Thus, the zebrafish is a well-suited model for further studies to elucidate the function of APOL1 in a living organism.

## Supporting Information

S1 Fig Breeding strategy. (TIF)

S2 Fig Confirmation of zApoL1 KD. (TIF)

S3 Fig zApoL1 by *in vivo* morpholinos. (TIF)

S4 Fig *in vivo* observation. (TIF)

S1 Movie 3D reconstruction of zApoL1 KD. (AVI)

S2 Movie 3 D reconstruction of CtrlMO. (AVI)

S3 Movie z-stacks of zApoL1 KD. (AVI)

S4 Movie z-stacks of zApoL1 CtrlMO. (AVI)

S1 Table (DOCX)
