# Supplementary figures and images for: Knockdown of ApoL1 in Zebrafish Larvae Affects the Glomerular Filtration Barrier and the Expression of Nephrin
Source: PLoS One. 2016 May 3;11(5):e0153768. doi: 10.1371/journal.pone.0153768 (PMC4854397; doi:10.1371/journal.pone.0153768)

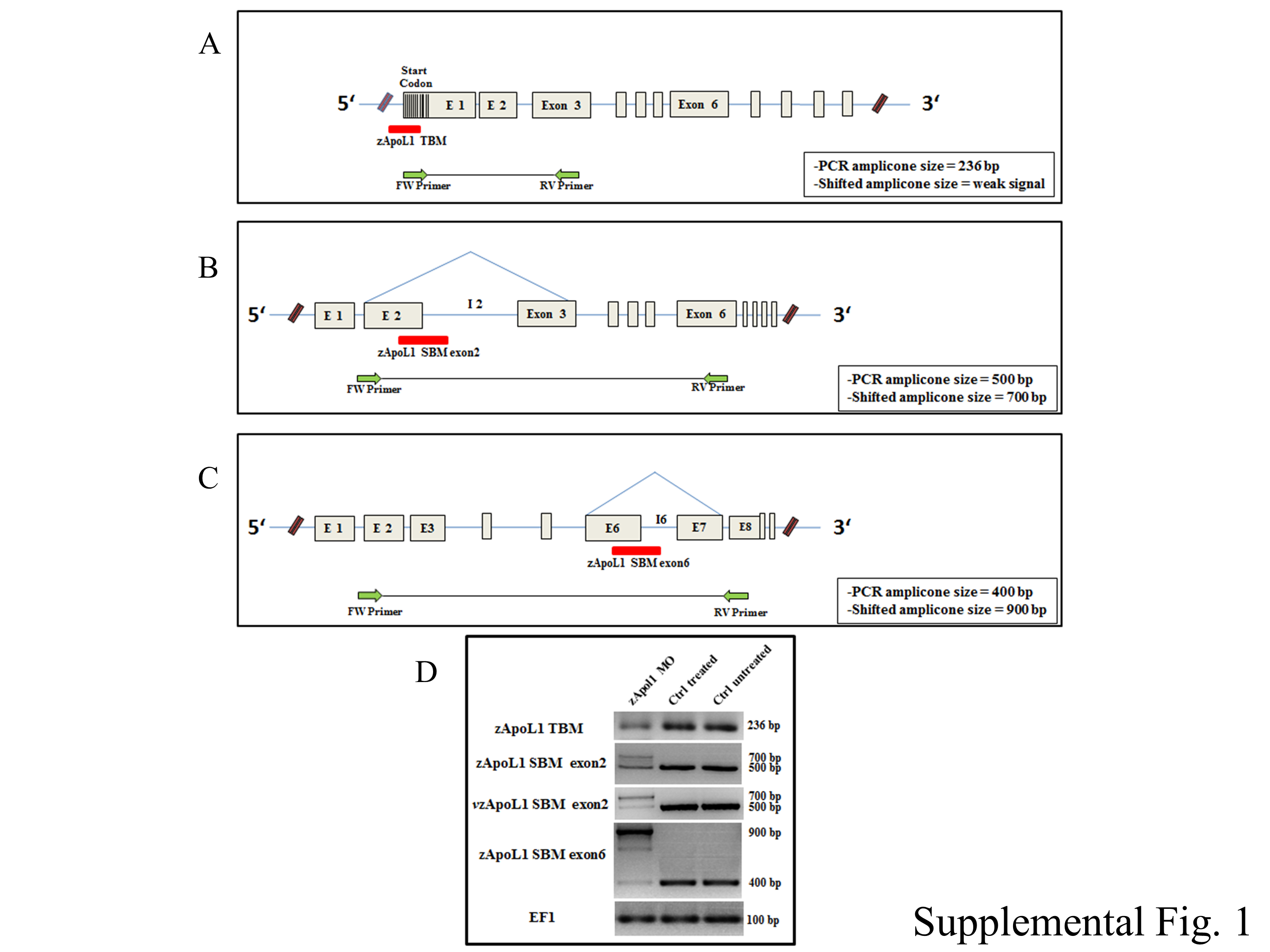

Supplement: S1 Fig — (TIF) [file pone.0153768.s001.TIF]

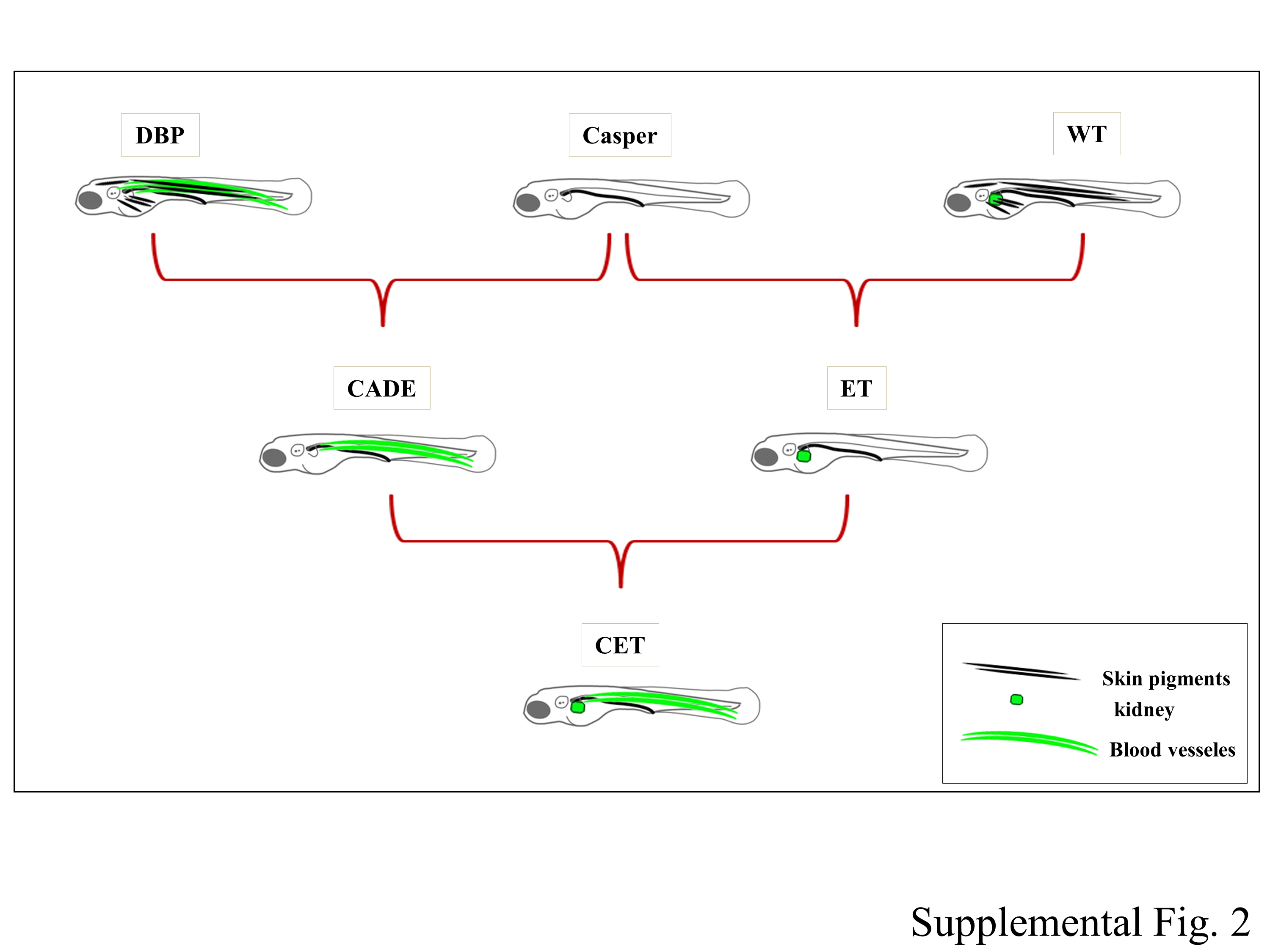

Supplement: S2 Fig — (TIF) [file pone.0153768.s002.TIF]

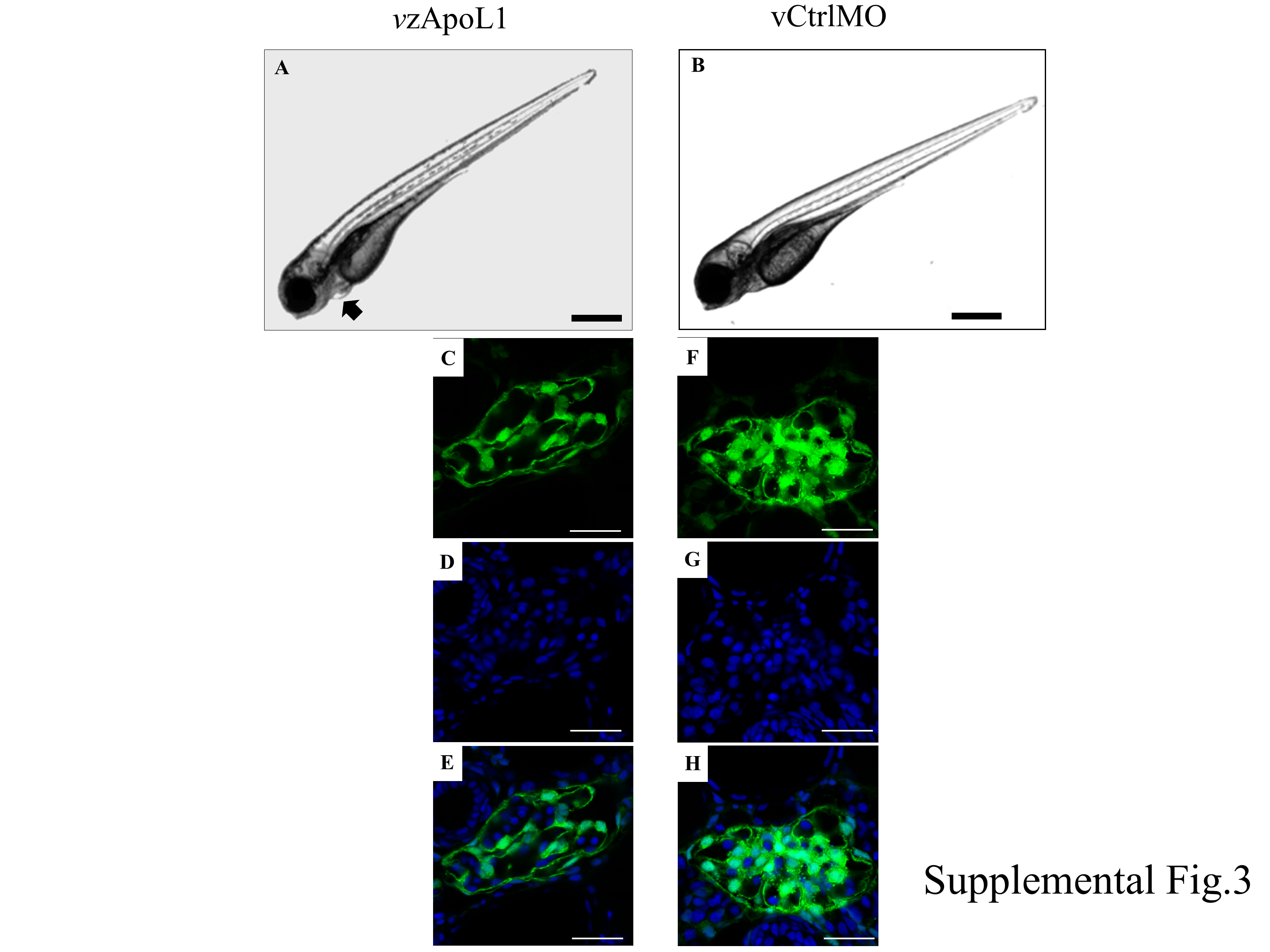

Supplement: S3 Fig — (TIF) [file pone.0153768.s003.TIF]

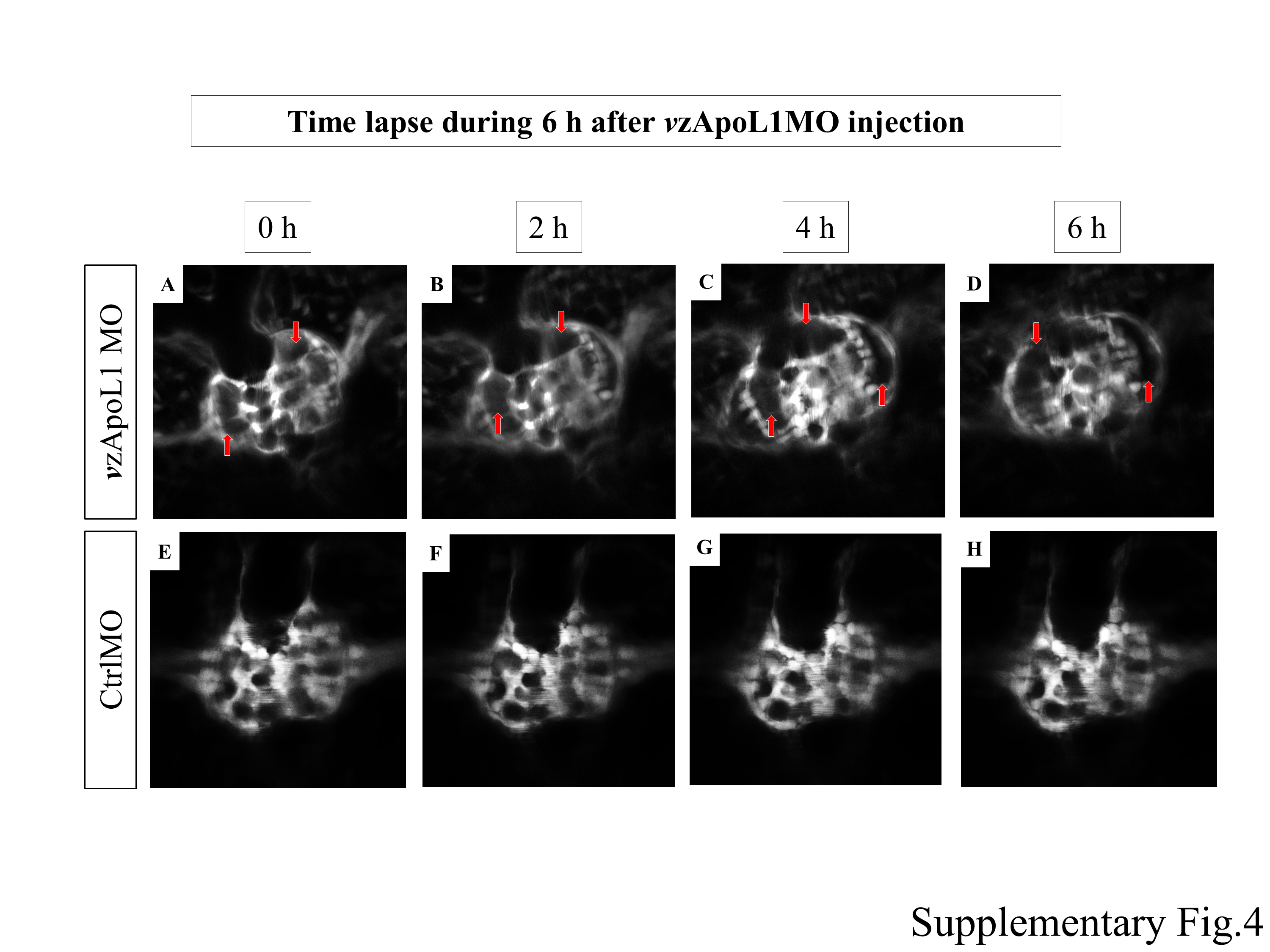

Supplement: S4 Fig — (TIF) [file pone.0153768.s004.TIF]
