# Supplementary material for: Knockdown of ApoL1 in Zebrafish Larvae Affects the Glomerular Filtration Barrier and the Expression of Nephrin
Source: PLoS One. 2016 May 3;11(5):e0153768. doi: 10.1371/journal.pone.0153768 (PMC4854397; doi:10.1371/journal.pone.0153768)
Supplement: S1 Table — (DOCX) [file pone.0153768.s009.docx]

S1 Table 1:

| zApoL1 fw | 5'-CTCATGGAATGGCTGGACGA-3' |
| --- | --- |
| zApoL1 rv | 5'-TAGCATGCAGGTCGATCACG-3' |
| zNephrin fw | 5'-GTCGTCCGAGCTTCGTGCCC-3' |
| zNephrin rv | 5'- TGCCAAGTGGGTCGGTCCGA-3' |
| EF1 fw | 5'- CTGGAGGCCAGCTCAAACAT-3' |
| EF1 rv | 5'- ATCAAGAAFAGTAGTACCGCTAGCATTAC-3' |
| hAPOL1 fw | 5'-GGTTCAGAAGGTCCACAAAG-3' |
| hAPOL1 rv | 5'-CTTGACTCCTCTGCTCATTTC-3' |
| hGAPDH fw | 5'- GAAGGTGAAGGTCGGAGTCAAC -3' |
| hGAPDH rv | 5'- CAGAGTTAAAAGCAGCCCTGGT -3' |
| zApoL1 SBM exon2 fw | 5'-TTGGAACCCAGTGAGAAAC-3' |
| zApoL1 SBM exon2 rv | 5'-ACACAAACTGCTCTTACTCTT-3' |
| zApoL1 SBM exon6 fw | 5'-TTGGAACCCAGTGAGAAAC-3' |
| zApol1 SBM exon6 rv | 5'-AAAGTCGTCCAGCCATTC-3' |
